# *Akkermansia muciniphila* and *Parabacteroides distasonis* as prognostic markers for relapse in ulcerative colitis patients

**DOI:** 10.3389/fcimb.2024.1367998

**Published:** 2024-07-04

**Authors:** Ana Mendes-Frias, Marta Moreira, Maria C. Vieira, Joana Gaifem, Patrício Costa, Luís Lopes, Ricardo Silvestre

**Affiliations:** ^1^ Life and Health Sciences Research Institute (ICVS), School of Medicine, University of Minho, Braga, Portugal; ^2^ ICVS/3B’s – PT Government Associate Laboratory, Braga/Guimarães, Portugal; ^3^ Department of Gastroenterology, Hospital Santa Luzia, Unidade Local de Saúde do Alto Minho, Viana do Castelo, Portugal; ^4^ i3S - Institute for Research and Innovation in Health, University of Porto, Porto, Portugal

**Keywords:** ulcerative colitis, *Akkermansia muciniphila*, *Parabacteroides distasonis*, disease relapse, inflammation

## Abstract

**Introduction:**

Ulcerative colitis is an inflammatory disorder characterized by chronic inflammation in the gastrointestinal tract, mainly in the colon and rectum. Although the precise etiology of ulcerative colitis remains unclear, recent research has underscored the significant role of the microbiome in its development and progression.

**Methods:**

The aim of this study was to establish a relationship between the levels of specific gut bacterial species and disease relapse in ulcerative colitis. For this study, we recruited 105 ulcerative colitis patients in remission and collected clinical data, blood, and stool samples. *Akkermansia muciniphila* and *Parabacteroides distasonis* levels were quantified in the stool samples of ulcerative colitis patients. Binary logistic regression was applied to collected data to predict disease remission.

**Results:**

The median time in remission in this cohort was four years. A predictive model incorporating demographic information, clinical data, and the levels of *Akkermansia muciniphila* and *Parabacteroides distasonis* was developed to understand remission patterns.

**Discussion:**

Our findings revealed a negative correlation between the levels of these two microorganisms and the duration of remission. These findings highlight the importance of the gut microbiota in ulcerative colitis for disease prognosis and for personalized treatments based on microbiome interventions.

## Introduction

Ulcerative Colitis (UC) is a chronic inflammatory disease that affects the colon and rectum, with a complex and multifactorial pathophysiology that involves environmental factors, immune system disorders and microbiome interactions in genetically susceptible individuals (Shen et al., 2018). UC has emerged as a global health challenge, owing to its elevated occurrence in developed countries and the progressively rising incidence in developing regions (Kobayashi et al., 2020). This increased incidence is justified by the adoption of a more westernized lifestyle, associated with increased consumption of highly processed and animal-based food (Wang et al., 2023). According to European Crohn´s and Colitis Organization (ECCO) guidelines, the current goal is to promote clinical remission defined as cessation of rectal bleeding and normal stool frequency, supported by increase of hemoglobin (Hb) and reduction of specific inflammatory markers (fecal calprotectin, erythrocyte sedimentation rate (ESR) and C reactive protein (CRP) (Travis et al., 2011; Raine et al., 2022). Ulcerative Colitis Symptoms Questionnaire (UC-SQ) is used to standardize symptoms characterization among clinical studies, allowing assessment of disease remission (Panes et al., 2023). After analysis, this scale was validated to evaluate responsiveness to treatment, and after modifications to a 15-point scale (excluding the joint pain and constipation) achieved an area under the curve (AUC) of 0.79 to estimate remission if the patient revealed 14 or less points in the questionnaire (Panes et al., 2023). Endoscopic and histological remission are determined based on colonoscopy with biopsies, through evaluation of the Mayo endoscopic sub-score (indicating remission if the score is 0–1) and mucosal lesions, respectively (Travis et al., 2011). Since the precise etiology of UC is not well-established, treatment strategies focus on achieving disease remission. The initial therapeutic approach involves the administration of aminosalicylates, while corticosteroids are used in relapse situations to mitigate the inflammatory response and tissue damage, thereby promoting restoration of homeostasis (Cai et al., 2021; Segal et al., 2021). Patients unresponsive to initial treatment require the administration of systemic immunomodulators such as biological agents. These agents consist of antibodies targeting key players of inflammatory response, primarily focusing on TNFα, α4β7-integrin and, more recently, IL-12/IL-23 (Raine, 2014; Genaro et al., 2021; Keam, 2023). Considering that all the therapies are administered to mitigate inflammation rather than provide a cure, and that these are only effective to a subset of patients, more research is needed to unravel specific targets to effectively manage UC.

Growing evidence has shown a significant contribution of microbiome to the development and maintenance of UC (Schirmer et al., 2018, 2024; Lloyd-Price et al., 2019; Jiang et al., 2024; Wu et al., 2024). Indeed, microbial species regulate immune function in the gut, inhibit proliferation and invasion of pathogenic agents, along with the production of metabolites with an important impact in intestinal homeostasis (Hu et al., 2022). Therefore, UC is characterized by a significant alteration in the composition of the microbiota (dysbiosis), with overgrowth of certain pathogenic bacteria and reduction of species with mucosal protective effects. These changes induce inflammation of the intestinal mucosa, compromising its barrier function, leading to an increased bacterial translocation, and promoting an exacerbated inflammatory response (Glassner et al., 2020). Previous work from our research group revealed a protective effect of *Akkermansia muciniphila* and *Parabacteroides distasonis* in murine models of chemically-induced colitis (Gaifem et al., 2024). The anti-inflammatory properties of *A. muciniphila* have been reported in both murine models and UC patients (Abbasi et al., 2023). However, the use of this strain as a probiotic requires further investigation since the beneficial impact of *A. muciniphila* depends on the strain used and, on the amount, administrated (Qu et al., 2023) (Liu et al., 2021). In addition, quantification of *A. muciniphila* have been reported in different cohorts, both adult and pediatric patients, reveling contradictory results (Chiantera et al., 2023; Zheng et al., 2023). Altogether, these studies revealed that the levels of *A. muciniphila* are altered by different factors, for instance diet and age, demonstrating that the use of *A. muciniphila* as a marker must be critically considered. *P. distasonis* was described to have a dichotomic effect in colitis, with reports revealing both anti and pro-inflammatory effect of this species (Ezeji et al., 2021). In addition, several reports revealed decreased levels of *P. distasonis* in UC patients during active disease (do Nascimento et al., 2020; Okahara et al., 2020; Nomura et al., 2021). However, the interaction between these two species is still under scrutiny.

In this work, we propose to evaluate the role of *A. muciniphila* and *P. distasonis* in UC relapse. A binary logistic regression that combines the clinical information of patients and the levels of *A. muciniphila* and *P. distasonis* was established to predict the interval of time for patients’ remission. We demonstrate an interaction between *A. muciniphila* and *P. distasonis* that is linked to a shorter time since relapse in patients experiencing remission.

## Materials and methods

### Patient admission and study design

**Figure 1:**
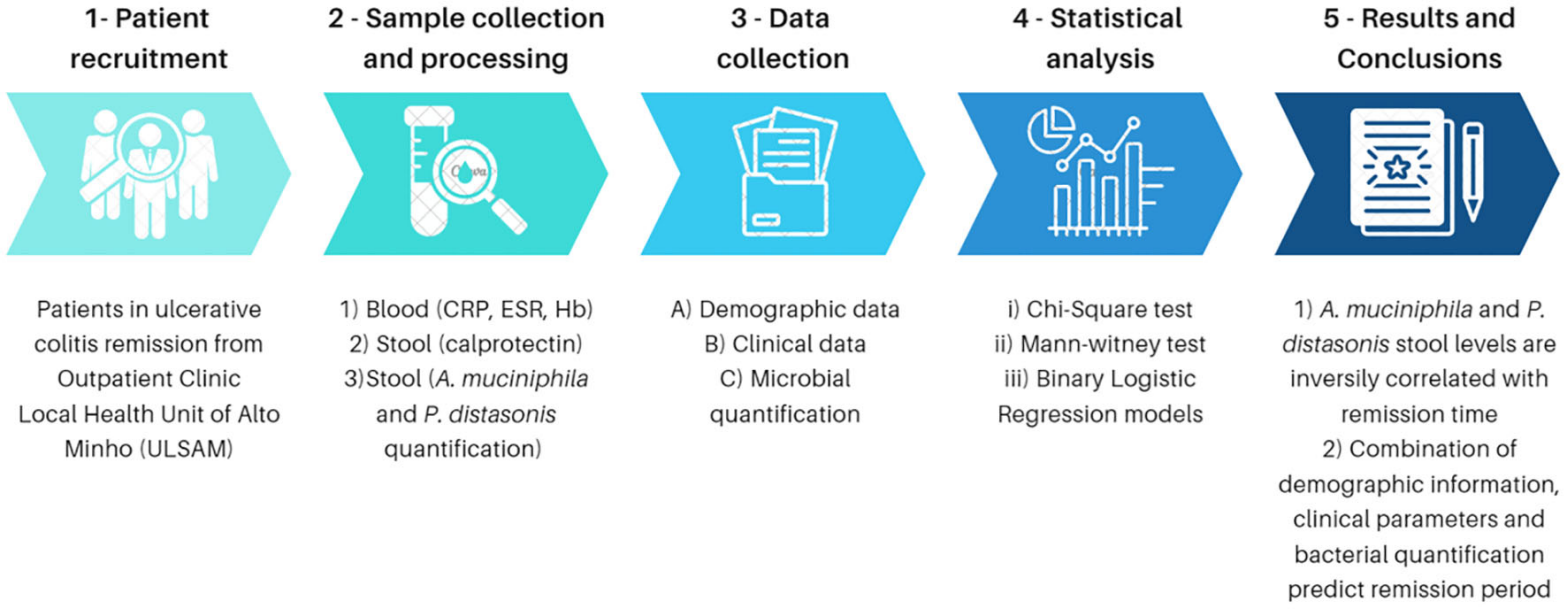
Study design.

This is a single-center prospective cohort study performed in collaboration between Life and Health Sciences Research Institute (ICVS) and Hospital de Santa Luzia [Local Health Unit of Alto Minho (ULSAM)], a Portuguese Hospital in Viana do Castelo affiliated with University of Minho summarized in **Figure 1**. From December 2021 to March 2023, all patients diagnosed with UC attending the Inflammatory Bowel Disease (IBD) outpatient clinic were invited to participate in this study. The study was approved by the local Ethic Committee of ULSAM with the reference 19/2020. Volunteer participants were informed about the aims of the study and signed an informed consent to participate. The study protocol conforms to the ethical guidelines of the 1975 Declaration of Helsinki as reflected in *a priori* approval by the institution’s human research committee. All patients with histologically confirmed UC followed at ULSAM with more than 18 years old were included. The exclusion criteria were patients who underwent colon resection surgery, had irregular follow-up appointments, had follow-up of less than one year or had taken antibiotics in the three months preceding sample collection.

From all the 105 participants, clinical data was collected using the UC-SQ to evaluate relevant symptoms that patients failed to report spontaneously, along with disease extension, time since last relapse, gender, age, age at onset, treatment, and use of antibiotics in the last three months. UC-SQ includes 17 different symptoms (frequency of bowel movements, passing of gases, abdominal pain, rectal pain, cramping, tiredness, loss of appetite, joint pain, difficulty sleeping, bloating, diarrhea, blood in stool, mucus in stool, constipation, false urge to defecate and defecatory urgency) rated from never to always regarding the 7 days prior to the questionnaire. During appointment, blood samples were collected to quantify inflammatory markers commonly used to follow UC patients: Hb, CRP and ESR. Finally, two sets of stool samples were collected; one to quantify fecal calprotectin and the other was immediately frozen until DNA extraction for quantification of the species of interest for this study. Samples added in multiple model 2 were longitudinal samples from the same patients already included, 6 months after the first sample.

The categorization of active disease or remission was determined through a medical evaluation that considered a combination of symptoms, analytical data, and colonoscopy findings. Symptoms were analysed using the UC-SQ relative to the seven days preceding the visit. Laboratory data collected during outpatient visits were defined as normal under the following criteria: Hb >12g/dL, CRP <1mg/dL, ESR <20mm/h, and fecal calprotectin <250μg/g. Remission was defined according to symptoms, biomarkers and latest endoscopy. However, in cases when these three parameters were not aligned, a rectosigmoidoscopy was performed to confirm remission. Only patients in disease remission were included in this study.

### DNA extraction

DNA from stool samples was extracted using Genomic DNA Kit (GK03.0100, Grisp) according to manufacturer’s instruction. A suggested protocol for DNA purification for stool samples was applied, using a lysozyme buffer (Lysozyme Buffer: 20mg/ml Lysozyme in 20mM Tris-HCl, 2mM EDTA, 1% Triton X-100) to efficiently lysate bacteria. Briefly, samples were homogenized using the lysozyme buffer and incubated with proteinase K (1mg/mL) and, then, RNase A (µg/mL) (both included in the kit). Samples were centrifuged at 16000g for 5minutes to remove insoluble components. DNA was isolated using a column included in the kit. After extraction, DNA was quantified using NanoDrop 1000 (Thermofisher).

### Bacterial quantification

Specific primers for *A. muciniphila* (CAGCACGTGAAGGTGGGGAC (forward) and CCTTGCGGTTGGCTTCAGAT (reverse)) and for *P. distasonis* (TGCCTATCAGAGGGGGATAAC (forward) and GCAAATATTCCCATGCGGGAT (reverse)) were used to obtain a specific sequence to each bacterium (Collado et al., 2007; Tong et al., 2011). This sequence was cloned in a vector using CloneJET PCR Cloning Kit (Thermo Scientific) to obtain a DNA template to each bacterium. A standard curve was obtained by qPCR using 10-fold dilutions of the template created. The bacterial abundance was determined by quantifying bacterial copy number in the DNA of stool samples using specific primers and the standard curve previously mentioned. Quantification results were normalized as number of copies per picograms of DNA.

### Statistical analysis

Qualitative variables were summarized using absolute and relative frequencies. The quantitative variables were summarized using the median and interquartile range, considering the small sample size and the non-normality observed in our variables, confirmed by normality tests (Kolmogorov-Smirnov and Shapiro-Wilk test) and histogram distribution.

The chi-square test was performed for categorical variables to assess the interdependence between variables. A Mann-Whitney test was employed to compare the study groups. For each statistical test we calculated the adequate effect size measure (r for the Mann-Whitney test and the V-Cramer/Phi for the chi-square test) and its interpretation followed the Cohen’s effect size cut-off values (Cohen, 2013). Binary logistic regressions models were employed and Odds Ratio (OR) and *p*-value were reported for each variable. Statistical analysis was performed using SPSS version 28 software (IBM, New York, USA).

## Results

### Demographic and clinical characterization of the cohort

Out of all the patients attending the appointments, 105 individuals diagnosed with UC and confirmed to be in a state of clinical disease remission as defined above were enrolled in this study. The demographic characterization of the cohort is presented in **Table 1**. Our gender-balanced cohort (48.6% males and 51.4% females) effectively invalidates gender-related influence in the subsequent analysis. Regarding endoscopic disease severity, patients included in this study exhibited endoscopic remission, indicated by a Mayo endoscopic sub-score of 0 and managed their symptoms with aminosalicylates (69.1% and 75.2%, respectively). Interestingly, laboratory parameters also indicated disease remission in our cohort comparing the median values with reference ranges, described in the Methods section. The median disease extension of our cohort is 12 years. The median time since relapse is 48 months (equivalent to 4 years). Consequently, we will consider the time since relapse as a categorical variable with two levels: exceeding 4 years and less than 4 years.

**Table 1:** Demographic and clinical characterization of the UC patients’ cohort (n=105).

Parameter		n or median (% or IQR)
Gender, n (%)	Males	51 (48.6)
	Females	54 (51.4)
Mayo score, n (%)	Score = 0	65 (61.9)
	Score = 1	40 (38.1)
Treatment	Aminosalicylates	79 (75.2)
	Biologics	26 (24.8)
Age, years		53 (20)
Age at onset, years		38 (55)
Remission Time, months		48 (58)
Disease extension, years		12 (18.5)
Hb, g/dL Remission: Hb>12g/dL		14.1 (7.10)
CRP, mg/dL Remission: CRP < 1mg/dL		0.15 (1.20)
ESR, mm/h Remission: ESR < 20 mm/h		12.0 (37.0)
Fecal Calprotectin, μg/g Remission: calprotectin < 250 μg/g		11.0 (27.0)

Hb, Hemoglobin; CRP, C Reactive Protein; ESR, Erythrocyte Sedimentation Rate; IQR, Interquartile Range.

Disease severity is often determined based on treatment, whereby patients unresponsive to the initial line of therapy (aminosalicylates) received biologics as a step-up approach (Cai et al., 2021). Following this criterion, patients are categorized as mild to moderate if treated with aminosalicylates, and as severe if they require biologics. To explore the potential impact of treatment on both clinical parameters and the levels of *A. muciniphila* and *P. distasonis*, this variable was employed as a grouping factor for comprehensive analysis (**Table 2**). No association was found between treatment and variables such as gender, age, time since relapse, Mayo score, disease extension and clinical parameters (Hb, ESR and fecal calprotectin) (*p*>0.05). However, when comparing patients on biologics to those taking aminosalicylates, the CRP levels were found significantly higher in the former group (*p*=0.022). Predominantly, the levels of *A. muciniphila* were undetectable in most patients (75%), aligning with existing literature indicating diminished abundance of this species in UC patients. Thus, the absolute abundance and the binary presence/absence of detection for *A. muciniphila* were considered. A similar approach was adopted for *P. distasonis*, although this species was quantified in most samples (83%). Ultimately, the analysis did not establish an association between the presence or combination of these species, according to the type of treatment, and subsequently, disease severity.

**Table 2:** Characterization of the different variables according to treatment (n=105).

Variable		Treatment		Statistical analysis		
		Aminosalicylates (n=79)	Biologics (n=26)	Statistic	*p* value	Effect Size**
Gender, n (%*)	Male	39 (37)	12 (11)	0.079	0.78	0.028
	Female	40 (38)	14 (14)			
Remission Time, n (%*)	More than 4 years	42 (40)	16 (15)	0.56	0.46	0.073
	Less than 4 years	37 (35)	10 (10)			
Mayo score, n (%*)	Score = 0	50 (48)	15 (14)	0.22	0.64	0.048
	Score = 1	29 (28)	11(10)			
*A. muciniphila*, n (%*)	Undetectable	59 (56)	20 (19)	0.053	0.82	0.022
	Detectable	20 (19)	6 (6)			
*P. distasonis*, n (%*)	Undetectable	12 (11)	6 (6)	0.86	0.36	0.090
	Detectable	67 (64)	20 (19)			
*A. muciniphila* * *P. distasonis*, n (%*)	Both undetected	12 (11)	6 (6)	0.86	0.65	0.093
	Pd detected	47 (45)	14 (13)			
	Both detected	20 (19)	6 (6)			
Age, median (IQR)		54 (21)	48 (18)	837	0.16	0.02
Age at onset, median (IQR)		39 (17)	33 (17)	884	0.29	0.011
Disease extension, median (IQR)		12 (19)	12 (21)	964	0.64	0.002
Hb, median (IQR) Remission: Hb>12g/dL		14.4 (1.83)	13.5 (1.38)	795	0.084	0.009
CRP, median (IQR) Remission: CRP < 1mg/dL		0.17 (0.23)	0.10 (0.13)	732	**0.022**	0.046
ESR, median (IQR) Remission: ESR < 20 mm/h		12 (8.25)	12 (8.25)	1097	0.61	0.002
Fecal Calprotectin, median (IQR) Remission: calprotectin < 250 μg/g		14 (29.5)	10 (17.3)	934	0.54	0.005
*A. muciniphila*, median (IQR) (copies/ug of DNA)		1.62 (13.7)	0.68 (39.2)	53	0.70	0.002
*P. distasonis*, median (IQR) (copies/ug of DNA)		13.4 (53)	15.9 (36.81)	670	0.99	0.001

Hb, Hemoglobin; CRP, C reactive Protein; ESR, Erythrocyte Sedimentation Rate; IQR, Interquartile Range.

*The percentage is calculated based on total sample size.

**For categorical variables Chi-square test was used to access the dependence of variables; effect size measures (Phi or Cramer’s V to 2x2 comparations or more, respectively) and p value are reported. For scale variables, Mann-Whitney test was employed; effect size measure (r) and p value are reported. Bold values are the p values with significance.

### *A. muciniphila* and *P. distasonis* detection is associated with a decrease time since relapse

Adopting a similar approach as for treatment type, we examined the association between the time since relapse, categorized in two intervals as previously mentioned (**Table 3**). No association was found between the demographic and clinical parameters and the time since relapse (*p*>0.05). However, in concordance with the literature, patients experiencing a relapse within the last 4 years were significantly younger than those who had not relapsed (*p*=0.011) (Jang et al., 2009; Nakov and Nakov, 2021). No significant differences emerged among the groups in the absolute levels of both *A. muciniphila* and *P. distasonis.* Interestingly, concerning the presence or absence of these bacteria, while the detection of *A. muciniphila* is not associated with the time since relapse, the presence of *P. distasonis* is significantly associated with decreased remission time (*p*=0.035), but with a small effect size (0.21). Remarkably, even when considering the detection of both species simultaneously, an association persisted between the presence of these bacteria and diminished duration of remission time (*p*=0.049).

**Table 3:** Characterization of the different variables according to time since relapse (n=105).

Variable		Remission Time		Statistical analysis		
		More than 4 years (n=58)	Less than 4 years (n=47)	Statistic	*p* value	Effect Size**
Gender, n (%*)	Male	26 (25)	25 (24)	0.73	0.40	0.08
	Female	32 (30)	22 (21)			
Treatment, n (%*)	Aminosalicylates	42 (40)	37 (35)	0.56	0.46	0.073
	Biologics	16 (15)	10 (10)			
Mayo score, n (%*)	Score = 0	39 (37)	26 (25)	1.12	0.29	0.11
	Score = 1	19 (18)	21 (20)			
*A. muciniphila*, n (%*)	Undetectable	43 (41)	36 (34)	0.084	0.77	0.028
	Detectable	15 (14)	11 (11)			
*P. distasonis*, n (%*)	Undetectable	14 (13)	4 (4)	4.46	**0.035**	0.21
	Detectable	44 (42)	43 (41)			
*A. muciniphila* * *P. distasonis*, n (%*)	Both undetectable	14 (13)	4 (4)	5.22	**0.049**	0.22
	Pd detected	29 (28)	32 (31)			
	Both detected	15 (14)	11 (10)			
Age, median (IQR)		54 (22)	46 (24)	969	**0.011**	0.061
Age at onset, median (IQR)		39 (18)	35 (16)	1118	0.11	0.024
Disease extension, median (IQR)		15 (19)	11 (15)	963	0.64	0.063
Hb, median (IQR) Remission: Hb>12g/dL		14.1 (1.75)	14.1 (1.83)	1316	0.76	0.001
CRP, median (IQR) Remission: CRP < 1mg/dL		0.17 (0.20)	0.13 (0.19)	1288	0.62	0.002
ESR, median (IQR) Remission: ESR < 20 mm/h		12 (10.3)	12 (7.00)	1364	0.99	0.0001
Fecal Calprotectin, median (IQR) Remission: calprotectin < 250 μg/g		11 (23.3)	14 (31.3)	1412	0.60	0.001
*A. muciniphila*, median (IQR) (copies/ug of DNA)		0.18 (4.0)	7.38 (13.7)	103	0.31	0.044
*P. distasonis*, median (IQR) (copies/ug of DNA)		11.9 (44.9)	17.4 (59.9)	1011	0.58	0.004

*The percentage is calculated based on total sample size.

**For categorical variables Chi-square test was used to assess the dependence of variables; effect size measures (Phi or Cramer’s V to 2x2 comparations or more, respectively) and p value are reported. For scale variables, Mann-Whitney test was employed; effect size measure (r) and p value are reported.

Hb, Hemoglobin; CRP, C reactive Protein; ESR, Erythrocyte Sedimentation Rate; IQR, Interquartile Range. Bold values are the p values with significance.

### *A. muciniphila* and *P. distasonis* levels enable prediction of remission time

**Figure 2:**
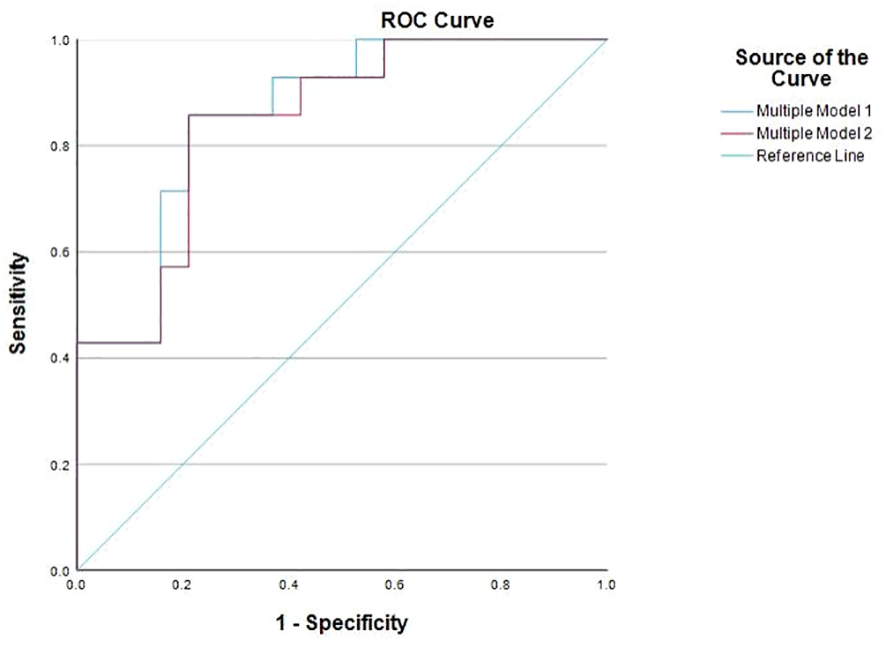
Receiver operating characteristic (ROC) curves of multiple model 1 and multiple model 2.

Predicting remission time in UC patients poses a significant challenge due to its dependency on many factors, including diet, daily habits (such as smoking and exercise) and microbiome composition. Given the observed relationship between the detection of *A. muciniphila* and *P. distasonis* and the time since relapse, we hypothesize that these two parameters might offer utility to predict the remission duration of UC patients. A binary logistic regression model was applied to all the variables as a single model to assess this. As presented in **Table 4**, in addition to age, which has already been pointed out as an important factor in disease remission, no other variable significantly impacted the time since relapse. Subsequently, an array of mixed models was assessed, encompassing a comprehensive spectrum of variables including clinical information and bacterial levels (absolute and detectable/undetectable). A significant and robust model (*p*=0.035 and R^2 =^ 0.53) was identified, including the following variables: gender, age, Hb, ESR, fecal calprotectin, the absolute levels of *A. muciniphila* and *P. distasonis* and the interaction between these latter two variables. However, only ESR revealed a significant contribution to the model (*p*=0.036). Subsequently, additional samples from the same patients at different time points were incorporated into the model to investigate whether the lack of significance was due to sample size constraints. Remarkably, with the inclusion of these supplementary samples, the model gained strength and significance (R^2 =^ 0.57 and *p*=0.001). Thus, the levels of the two species under analysis and their interaction began to influence the prediction of the remission time significantly. This concerned not only individual species but also the combination of both (*p*=0.026 to *A. muciniphila*, *p*=0.046 to *P. distasonis* and *p=*0.026 for the interaction). Of note, in both models, the adjusted ORs for *A. muciniphila, P. distasonis* and their interaction remained consistent (OR=1.58, 1.01 and 0.99 to *A. muciniphila, P. distasonis* and their interaction, respectively, in the first model and OR=1.87, 1.01 and 0.99, respectively, in the second model), meaning that the contribution of each variable is the same in both models. A receiver operating characteristic (ROC) curve was created to each mixed model (**Figure 2**). An area under the curve (AUC) = 0.86 (CI 95%: 0.74;0.99, *p*<0.0001) and AUC = 0.846 (CI:0.71;0.98, *p*=0.001) was obtained to model 1 and model 2, respectively. Consequently, it can be concluded that the levels of *A. muciniphila* and *P. distasonis* indeed contribute to determining the time since relapse in UC patients.

**Table 4:** Binary logistic regression to predict remission time.

Variables		Simple model				Multiple model 1 (n=33) (*p* value=0.035; R^2^ = 0.53)			Multiple model 2 (n=46) (*p* value=0.001; R^2^ = 0.57)		
		OR	*p* value	R^2^	95% CI for Exp (B)	Adjusted OR	*p* value	95% CI for Exp (B)	Adjusted OR	*p* value	95% CI for Exp (B)
**Nominal**	Gender	0.74	0.45	0.007	0.35 – 1.60	0.90	0.93	0.083 – 9.80	1.29	0.81	0.16 – 10.34
	Medication	0.64	0.34	0.010	0.26 – 1.58	—	—	—	—	—	—
	Mayo Score	1.57	0.13	0.050	0.87 – 2.84	—	—	—	—	—	—
	*A. muciniphila*	0.79	0.61	0.003	0.32 – 1.94	—	—	—	—	—	—
	*P. distasonis*	1.96	0.22	0.020	0.67 – 2.68	—	—	—	—	—	—
	A. muciniphila * P. distasonis	1.13	0.70	0.002	0.62 – 2.04	—	—	—	—	—	—
**Scale**	Age	0.96	**0.004**	0.11	0.93 – 0.99	0.90	0.13	0.79 – 1.03	0.86	**0.016**	0.76 – 0.97
	Age at onset	0.97	0.091	0.037	0.94 – 1.00	—	—	—	—	**—**	—
	Disease extension	0.97	0.11	0.033	0.94 – 1.01	—	—	—	—	—	—
	Hb	0.97	0.84	0.001	0.73 – 1.30	1.97	0.33	0.51 – 7.70	3.75	0.078	0.86 – 16.26
	CRP	0.79	0.63	0.003	0.31 – 2.05	—	—	—	—	—	—
	ESR	1.00	0.91	0.001	0.95 – 1.04	1.46	**0.036**	1.03 – 2.06	1.65	**0.005**	1.16 – 2.34
	Fecal Calprotectin	1.01	0.11	0.057	0.99 – 1.01	0.99	0.68	0.93 – 1.05	1.00	0.13	0.99 – 1.01
	*A. muciniphila*	0.99	0.39	0.056	0.96 – 1.02	1.58	0.096	0.92 – 2.72	1.87	**0.026**	1.08 – 3.25
	*P. distasonis*	1.00	0.89	0.001	0.99 – 1.00	1.01	0.13	0.99 – 1.01	1.01	**0.046**	1.00 – 1.02
	A. muciniphila * P. distasonis	1.00	0.69	0.10	0.99 – 1.00	0.99	0.11	0.98 – 1.00	0.99	**0.026**	0.98 – 1.00

Hb, Hemoglobin, CRP; C reactive Protein; ESR, Erythrocyte Sedimentation Rate; Odds Ratio, OR; Confidence Interval, CI. Bold values are the p values with significance. * means interaction between the two variables.

## Discussion

UC has emerged as a global burden, affecting millions worldwide, with an increasing incidence over the last few years (Wang et al., 2023). While the precise etiology of the disease remains elusive, significant efforts have been made to define accurate diagnostic and monitoring markers (Feuerstein et al., 2019). The intestinal microbiome plays a crucial role in the gut homeostasis and the development and progression of UC. Given the consistent alterations in the microbiome composition in UC patients, identifying microbial markers may be pivotal for a more effective clinical management of the disease (Glassner et al., 2020). The quantification of these microbial markers or even the microorganisms themselves, is valuable for enhancing diagnosis, improving patient follow-up as a prognostic marker, and predicting treatment response and disease recurrence in a non-invasive manner (Guo et al., 2022).

The patient cohort evaluated in this study was balanced for age and gender, in which all patients were classified as being in remission state of the disease. Regarding treatment, in our cohort 75% of patients maintain disease remission only with aminosalicylates therapy (mesalazine), while 25% of patients require biologic agents (infliximab, adalimumab, ustekinumab or vedolizumab) to achieve remission. These percentages are consistent with European studies, particularly with a Spanish cohort that reported a remission maintenance rate of 72% among UC patients treated with aminosalicylates (Marti-Aguado et al., 2019).

Clinical parameters have been used as follow-up measures for disease activity in UC (Sakurai and Saruta, 2023). Reduced levels of fecal calprotectin have been identified as a predictive indicator for mucosal healing and colitis remission, establishing it as a non-invasive marker of remission (Malvão et al., 2021; Kawashima et al., 2023). However, our study did not find statistically significant differences in fecal calprotectin levels between the two remission groups. Notably, a median of 11 μg/g was observed for patients in remission for over 4 years, in contrast to a median of 14 μg/g for patients with a remission period lower than 4 years. Also, the predictive value of fecal calprotectin was not evident in our study, either alone (*p* = 0.11) or in combination with the other markers (*p* = 0.68 to the first model and *p* = 0.13). While most studies compare remission and active disease, our study establish a four-year remission cut-off, indicating fecal calprotectin’s limitations in discriminating based on the duration of remission. In contrast, our study unveiled a significant association between ESR levels and the prediction of remission period. ESR, a classic inflammatory marker widely used for diagnosis and follow-up in UC patients, despite its elevation in various inflammatory conditions (Sakurai and Saruta, 2023), ESR has been suggested by other studies as predictor of recurrence in UC, comparing active disease with remission (Liverani et al., 2016). Our findings indicate that ESR serves as a complementary marker for predicting the remission period. When used alone in a binary logistic model, ESR did not show predictive value; however, its significance emerged when combined with several markers in predicting disease remission.

In this work, we propose two microbial targets, *A. muciniphila* and *P. distasonis*, as specific markers for disease remission. Both bacteria have been associated with reports of both beneficial and pathogenic effects on human health (Ezeji et al., 2021; Abbasi et al., 2023). Additionally, recently published data from our research group suggests an interaction between these two species associated with a protective phenotype in mouse models of UC (Gaifem et al., 2024). Our findings suggest an inverse correlation between the levels of these species and remission time. Indeed, we have established a robust and significant model that combines the levels of these two microorganisms with clinical parameters to predict the remission time. Additionally, our data suggest an interaction between the levels of *A. muciniphila* and *P. distasonis* and that these two species directly impact remission period in UC patients.

*A. muciniphila*, a gram-negative anaerobe, resides in the mucus layer and degrades mucin to produce metabolites (short-chain fatty acids) that benefit the intestinal epithelial barrier (Derrien et al., 2004). Indeed, an increased abundance of these bacteria has been reported in healthy individuals, while decreased levels were found in UC patients (Derrien et al., 2008). In recent years, the beneficial properties of this species have been extensively explored in mouse models of UC, suggesting *A. muciniphila* as a potential candidate for probiotic use as an adjuvant to therapy (Abbasi et al., 2023; Zheng et al., 2023). However, excessive colonization of *A. muciniphila* promotes increased degradation of mucins from the mucus layer, leading to the concomitant destruction of the epithelial barrier structure and inflammation in the gut (Qu et al., 2023). Indeed, the effect of *A. muciniphila* in a DSS-induced colitis mouse model was demonstrated to depend on the administered strain (Liu et al., 2021). In our cohort, no significant association was found between the levels of *A. muciniphila* and remission time. Herrera-deGuise and colleagues found a positive correlation between the levels of this microorganism and remission time (Herrera-deGuise et al., 2023). These variations may be attributed to the different analysis performed in both studies. In our study, only samples above the detection limit were included in the analysis, which reduced considerably the sample size (*A. muciniphila* was only detectable in 25% of all samples). To overcome this limitation, we created a dichotomic variable referring to the presence or absence of both species, so that all samples could be included. The Spanish study used a method in which values below the detection limit were treated by imputing them as the lowest abundance in the data set divided by 2. While this approach offers the advantage of incorporating all data points into subsequent analyses, it’s crucial to note that this imputation was applied in a relevant number of cases, leading to a reduction in the dispersion of the variable distribution. Thus, the discrepancy in results could be explained by this increase in the number of patients with low levels of *A. muciniphila*.

*P. distasonis*, a gram negative aerotolerant bacterium, colonizes the gastrointestinal tract and exhibit a dichotomous effect on the host, with both pathogenic and beneficial effects (Ezeji et al., 2021). On one hand, *P. distasonis* administration alleviates obesity, metabolic dysfunctions, and experimental colitis, in both DSS and TNBS mouse models (Kverka et al., 2011; Wang et al., 2019; Cuffaro et al., 2020). On the other hand, the abundance of this microorganism has been found to increase in acute and chronic DSS-induced colitis model, promoting inflammation in mouse models by degrading the mucosal barrier, potentially contributing to the development of intestinal inflammation (Okayasu et al., 1990; González-Páez et al., 2019). Additionally, a dichotomous effect of this microorganism has been observed in the context of diabetes: while Cai and colleagues demonstrate the anti-inflammatory properties of *P. distasonis* that could prevent diabetes by decreasing insulin resistance, Hasain and colleagues found an enrichment of this species in gestational diabetes mellitus (Cai et al., 2020; Hasain et al., 2020). Also, the levels of this microorganism are also inversely correlated with the presence of intestinal tumors, emphasizing that the influence of *P. distasonis* levels in disease varies in a context-dependent manner (Kverka et al., 2011; Wang et al., 2019; Cuffaro et al., 2020; Gu et al., 2021). Our work found an association between the levels of *P. distasonis* and disease remission, with an increased level of this microorganism in individuals with a remission time lower than four years. Overall, these data suggest that *P. distasonis* may contribute for the immunopathological events underlying remission of UC. However, more research is needed to understand the mechanisms underlying this impact.

In this study, we integrated patient demographic, clinical and microbial data to create a predictive model for remission time. Our model reveals that higher levels of *A. muciniphila* and *P. distasonis* and their interaction are associated with decreased remission time. This is the first association between these two microorganisms in the context of UC, specifically within a patient cohort. Despite the small sample size and the challenges to quantify *A. muciniphila*, our model is an important clinical tool for predicting UC patients’ remission time due to its lower levels in UC patients. Given the evident influence of sample size on *p*-values, it is advisable to closely monitor and discuss the effect size measures, namely OR in this case. Although we did not initially obtain statistically significant results for these two microorganisms (and their interaction), the calculated OR values suggest that even a modest increase in our sample size could potentially yield significant results. Additionally, a R^2^ greater than 0.5 reflects that this is a robust model for predicting remission time, according to Cohen’s cut-off values (Cohen, 2013). Thus, the extension of our cohort could increase statistical power of the tested models. Also, the integration of Crohn’s patients in our cohort could benefit the knowledge about the different forms of IBD and the influence of the microbiome in both diseases. Nevertheless, this study demonstrates a novel promising model that can provide relevant information for UC clinical management, since it can be used to infer relapses in patients that show higher risk for remission. The evaluation of specific bacterial species (in this case, *A. muciniphila* and *P. distasonis*), in combination with demographic parameters and biochemical markers can constitute an important tool to predict remission, as previously defined by other research groups (Hosseini et al., 2015; Pang et al., 2023).

In conclusion, our results have enhanced the understanding of microbial changes in the Portuguese population during UC remission. Considering that the microbiome varies significantly across populations due to differences in diet and lifestyle, these cohort studies are crucial to characterize populations fully and should take into account the changes in diet between individuals, that were not considered in this work. Smoking habits have also been described to shape the microbiome and to affect UC onset (Papoutsopoulou et al., 2020; Gui et al., 2021; Nicolaides et al., 2021). However, the smoking habits of the participants were not available. Nevertheless, understanding which and how different microbial players interact with these two species may allow the improvement of microbiota-targeted therapies, tailored to different subsets of patients. This ultimately will contribute towards a more personalized healthcare approach and an improved quality of life for UC patients.

## Data availability statement

The raw data supporting the conclusions of this article will be made available by the authors, without undue reservation.

## Ethics statement

The study was approved by the local Ethic Committee of Local Health Unit of Alto Minho (ULSAM) with the reference 19/2020. The studies were conducted in accordance with the local legislation and institutional requirements. The participants provided their written informed consent to participate in this study. Written informed consent was obtained from the individual(s) for the publication of any potentially identifiable images or data included in this article.

## Author contributions

AF: Writing – original draft, Writing – review & editing, Conceptualization, Data curation, Formal analysis, Investigation, Methodology. MM: Data curation, Formal analysis, Investigation, Methodology, Writing – review & editing. MV: Formal analysis, Investigation, Methodology, Writing – review & editing. JG: Conceptualization, Data curation, Writing – review & editing. PC: Data curation, Formal analysis, Writing – review & editing, Methodology. LL: Project administration, Resources, Supervision, Validation, Writing – review & editing. RS: Conceptualization, Funding acquisition, Project administration, Resources, Supervision, Validation, Writing – review & editing.

## References

[B1] Abbasi A. Bazzaz S. Da Cruz A. G. Khorshidian N. Saadat Y. R. Sabahi S.. (2023). A critical review on akkermansia muciniphila: functional mechanisms, technological challenges, and safety issues. Probiotics Antimicrob. Proteins. 1. doi: 10.1007/s12602-023-10118-x 37432597

[B2] Cai W. Xu J. Li G. Liu T. Guo X. Wang H.. (2020). Ethanol extract of propolis prevents high-fat diet-induced insulin resistance and obesity in association with modulation of gut microbiota in mice. Food Res. Int. 130, 108939. doi: 10.1016/j.foodres.2019.108939 32156386

[B3] Cai Z. Wang S. Li J. (2021). Treatment of inflammatory bowel disease: A comprehensive review. Front. Med. 8. doi: 10.3389/fmed.2021.765474 PMC8720971 34988090

[B4] Chiantera V. Laganà A. S. Basciani S. Nordio M. Bizzarri M. (2023). A critical perspective on the supplementation of akkermansia muciniphila: benefits and harms. Life 13, 1247. doi: 10.3390/life13061247 37374030 PMC10301191

[B5] Cohen J. (2013). Statistical power analysis for the behavioral sciences (Cambridge, Academic Press: Routledge). doi: 10.4324/9780203771587

[B6] Collado M. C. Derrien M. Isolauri E. de Vos W. M. Salminen S. (2007). Intestinal integrity and akkermansia muciniphila, a mucin-degrading member of the intestinal microbiota present in infants, adults, and the elderly. Appl. Environ. Microbiol. 73, 7767–7770. doi: 10.1128/AEM.01477-07 17933936 PMC2168041

[B7] Cuffaro B. Assohoun A. L. W. Boutillier D. Súkeníková L. Desramaut J. Boudebbouze S.. (2020). *In vitro* characterization of gut microbiota-derived commensal strains: selection of parabacteroides distasonis strains alleviating TNBS-induced colitis in mice. Cells 9, 2104. doi: 10.3390/cells9092104 32947881 PMC7565435

[B8] Derrien M. Collado M. C. Ben-Amor K. Salminen S. de Vos W. M. (2008). The mucin degrader *akkermansia muciniphila* is an abundant resident of the human intestinal tract. Appl. Environ. Microbiol. 74, 1646–1648. doi: 10.1128/AEM.01226-07 18083887 PMC2258631

[B9] Derrien M. Vaughan E. E. Plugge C. M. de Vos W. M. (2004). Akkermansia muciniphila gen. nov., sp. nov., a human intestinal mucin-degrading bacterium. Int. J. Syst. Evol. Microbiol. 54, 1469–1476. doi: 10.1099/ijs.0.02873-0 15388697

[B10] do Nascimento R.de P. MaChado A.P.da F. Galvez J. Cazarin C. B. B. Maróstica Junior M. R. (2020). Ulcerative colitis: Gut microbiota, immunopathogenesis and application of natural products in animal models. Life Sci. 258, 1–22. doi: 10.1016/j.lfs.2020.118129 32717271

[B11] Ezeji J. C. Sarikonda D. K. Hopperton A. Erkkila H. L. Cohen D. E. Martinez S. P.. (2021). Parabacteroides distasonis: intriguing aerotolerant gut anaerobe with emerging antimicrobial resistance and pathogenic and probiotic roles in human health. Gut Microbes 13, 1–27. doi: 10.1080/19490976.2021.1922241 PMC8253142 34196581

[B12] Feuerstein J. D. Moss A. C. Farraye F. A. (2019). Ulcerative colitis. Mayo Clin. Proc. 94, 1357–1373. doi: 10.1016/j.mayocp.2019.01.018 31272578

[B13] Gaifem J. Mendes-Frias A. Wolter M. Steimle A. Garzón M. J. Ubeda C.. (2024). Akkermansia muciniphila and Parabacteroides distasonis synergistically protect from colitis by promoting ILC3 in the gut. mBio. 15. doi: 10.1128/mbio.00078-24 PMC11210198 38470269

[B14] Genaro L. M. Gomes L. E. M. Franceschini A. P. M. de F. Ceccato H. D. de Jesus R. N.. (2021). Anti-TNF therapy and immunogenicity in inflammatory bowel diseases: a translational approach. Am. J. Transl. Res. 13, 13916–13930. 35035733 PMC8748125

[B15] Glassner K. L. Abraham B. P. Quigley E. M. M. (2020). The microbiome and inflammatory bowel disease. J. Allergy Clin. Immunol. 145, 16–27. doi: 10.1016/j.jaci.2019.11.003 31910984

[B16] González-Páez G. E. Roncase E. J. Wolan D. W. (2019). X-ray structure of an inactive zymogen clostripain-like protease from *Parabacteroides distasonis* . Acta Crystallogr. D Struct. Biol. 75, 325–332. doi: 10.1107/S2059798319000809 30950403

[B17] Gu Z.-Y. Pei W.-L. Zhang Y. Zhu J. Li L. Zhang Z. (2021). Akkermansia muciniphila in inflammatory bowel disease and colorectal cancer. Chin. Med. J. (Engl) 134, 2841–2843. doi: 10.1097/CM9.0000000000001829 34711719 PMC8667969

[B18] Gui X. Yang Z. Li M. D. (2021). Effect of cigarette smoke on gut microbiota: state of knowledge. Front. Physiol. 12. doi: 10.3389/fphys.2021.673341 PMC8245763 34220536

[B19] Guo X. Huang C. Xu J. Xu H. Liu L. Zhao H.. (2022). Gut microbiota is a potential biomarker in inflammatory bowel disease. Front. Nutr. 8. doi: 10.3389/fnut.2021.818902 PMC8814525 35127797

[B20] Hasain Z. Mokhtar N. M. Kamaruddin N. A. Mohamed Ismail N. A. Razalli N. H. Gnanou J. V.. (2020). Gut microbiota and gestational diabetes mellitus: A review of host-gut microbiota interactions and their therapeutic potential. Front. Cell Infect. Microbiol. 10. doi: 10.3389/fcimb.2020.00188 PMC7243459 32500037

[B21] Herrera-deGuise C. Varela E. Sarrabayrouse G. Pozuelo del Río M. Alonso V. R. Sainz N. B.. (2023). Gut microbiota composition in long-remission ulcerative colitis is close to a healthy gut microbiota. Inflammation Bowel Dis. 29, 1362–1369. doi: 10.1093/ibd/izad058 37655859

[B22] Hosseini S. V. Safarpour A. R. Taghavi S. A. (2015). Developing a novel risk-scoring system for predicting relapse in patients with ulcerative colitis: A prospective cohort study. Pak J. Med. Sci. 31, 1511–1516. doi: 10.12669/pjms.316.8811 26870126 PMC4744311

[B23] Hu Y. Chen Z. Xu C. Kan S. Chen D. (2022). Disturbances of the gut microbiota and microbiota-derived metabolites in inflammatory bowel disease. Nutrients 14, 5140. doi: 10.3390/nu14235140 36501169 PMC9735443

[B24] Jang E. S. Lee D. H. Kim J. Yang H.-J. Lee S. H. Park Y. S.. (2009). Age as a clinical predictor of relapse after induction therapy in ulcerative colitis. Hepatogastroenterology 56, 1304–1309. 19950781

[B25] Jiang N. Liu Z. Wang H. Zhang L. Li M. Li G.. (2024). Alterations in metabolome and microbiome: new clues on cathelicidin-related antimicrobial peptide alleviates acute ulcerative colitis. Front. Microbiol. 15. doi: 10.3389/fmicb.2024.1306068 PMC10877057 38380090

[B26] Kawashima K. Oshima N. Kishimoto K. Kataoka M. Fukunaga M. Kotani S.. (2023). Low fecal calprotectin predicts histological healing in patients with ulcerative colitis with endoscopic remission and leads to prolonged clinical remission. Inflammation Bowel Dis. 29, 359–366. doi: 10.1093/ibd/izac095 35583193

[B27] Keam S. J. (2023). Mirikizumab: first approval. Drugs 83, 1045–1052. doi: 10.1007/s40265-023-01909-1 37389706

[B28] Kobayashi T. Siegmund B. Le Berre C. Wei S. C. Ferrante M. Shen B.. (2020). Ulcerative colitis. Nat. Rev. Dis. Primers 6, 1–20. doi: 10.1038/s41572-020-0205-x 32913180

[B29] Kverka M. Zakostelska Z. Klimesova K. Sokol D. Hudcovic T. Hrncir T.. (2011). Oral administration of Parabacteroides distasonis antigens attenuates experimental murine colitis through modulation of immunity and microbiota composition. Clin. Exp. Immunol. 163, 250–259. doi: 10.1111/j.1365-2249.2010.04286.x 21087444 PMC3043316

[B30] Liu Q. Lu W. Tian F. Zhao J. Zhang H. Hong K.. (2021). Akkermansia muciniphila exerts strain-specific effects on DSS-induced ulcerative colitis in mice. Front. Cell Infect. Microbiol. 11. doi: 10.3389/fcimb.2021.698914 PMC8371549 34422681

[B31] Liverani E. Scaioli E. Digby R. J. Bellanova M. Belluzzi A. (2016). How to predict clinical relapse in inflammatory bowel disease patients. World J. Gastroenterol. 22, 1017. doi: 10.3748/wjg.v22.i3.1017 26811644 PMC4716017

[B32] Lloyd-Price J. Arze C. Ananthakrishnan A. N. Schirmer M. Avila-Pacheco J. Poon T. W.. (2019). Multi-omics of the gut microbial ecosystem in inflammatory bowel diseases. Nature 569, 655–662. doi: 10.1038/s41586-019-1237-9 31142855 PMC6650278

[B33] Malvão L. dos R. Madi K. Esberard B. C. de Amorim R. F. Silva K. dos S.. (2021). Fecal calprotectin as a noninvasive test to predict deep remission in patients with ulcerative colitis. Medicine 100, e24058. doi: 10.1097/MD.0000000000024058 33546007 PMC7837839

[B34] Marti-Aguado D. Ballester M. P. Tosca J. Bosca-Watts M. M. Navarro P. Anton R.. (2019). Long-term follow-up of patients treated with aminosalicylates for ulcerative colitis: Predictive factors of response: An observational case-control study. United Eur. Gastroenterol. J. 7, 1042–1050. doi: 10.1177/2050640619854277 PMC6794696 31662861

[B35] Nakov R. Nakov V. (2021). Young age and short duration of the disease are associated with more frequent relapses in inflammatory bowel disease patients. Med. Pharm. Rep. 94, 43–47. doi: 10.15386/mpr-1510 33629047 PMC7880070

[B36] Nicolaides S. Vasudevan A. Long T. van Langenberg D. (2021). The impact of tobacco smoking on treatment choice and efficacy in inflammatory bowel disease. Intest Res. 19, 158–170. doi: 10.5217/ir.2020.00008 33040518 PMC8100381

[B37] Nomura K. Ishikawa D. Okahara K. Ito S. Haga K. Takahashi M.. (2021). Bacteroidetes species are correlated with disease activity in ulcerative colitis. J. Clin. Med. 10, 1749. doi: 10.3390/jcm10081749 33920646 PMC8073534

[B38] Okahara K. Ishikawa D. Nomura K. Ito S. Haga K. Takahashi M.. (2020). Matching between donors and ulcerative colitis patients is important for long-term maintenance after fecal microbiota transplantation. J. Clin. Med. 9, 1650. doi: 10.3390/jcm9061650 32486476 PMC7355579

[B39] Okayasu I. Hatakeyama S. Yamada M. Ohkusa T. Inagaki Y. Nakaya R. (1990). A novel method in the induction of reliable experimental acute and chronic ulcerative colitis in mice. Gastroenterology 98, 694–702. doi: 10.1016/0016-5085(90)90290-H 1688816

[B40] Panes J. Otley A. Sanchez Gonzalez Y. Fitzgerald K. Zhou W. Ilo D.. (2023). Ulcerative colitis-symptom questionnaire: valid for use in adults with moderately to severely active ulcerative colitis. Dig Dis. Sci. 68, 2318–2332. doi: 10.1007/s10620-022-07807-y 36773193 PMC10188579

[B41] Pang W. Zhang B. Jin L. Yao Y. Han Q. Zheng X. (2023). Serological biomarker-based machine learning models for predicting the relapse of ulcerative colitis. J. Inflammation Res. Volume 16, 3531–3545. doi: 10.2147/JIR.S423086 PMC10455884 37636275

[B42] Papoutsopoulou S. Satsangi J. Campbell B. J. Probert C. S. (2020). Review article: impact of cigarette smoking on intestinal inflammation—direct and indirect mechanisms. Aliment Pharmacol. Ther. 51, 1268–1285. doi: 10.1111/apt.15774 32372449

[B43] Qu S. Zheng Y. Huang Y. Feng Y. Xu K. Zhang W.. (2023). Excessive consumption of mucin by over-colonized Akkermansia muciniphila promotes intestinal barrier damage during Malignant intestinal environment. Front. Microbiol. 14. doi: 10.3389/fmicb.2023.1111911 PMC10018180 36937258

[B44] Raine T. (2014). Vedolizumab for inflammatory bowel disease: Changing the game, or more of the same? United Eur. Gastroenterol. J. 2, 333–344. doi: 10.1177/2050640614550672 PMC4212504 25360311

[B45] Raine T. Bonovas S. Burisch J. Kucharzik T. Adamina M. Annese V.. (2022). ECCO guidelines on therapeutics in ulcerative colitis: medical treatment. J. Crohns Colitis 16, 2–17. doi: 10.1093/ecco-jcc/jjab178 34635919

[B46] Sakurai T. Saruta M. (2023). Positioning and usefulness of biomarkers in inflammatory bowel disease. Digestion 104, 30–41. doi: 10.1159/000527846 36404714 PMC9843547

[B47] Schirmer M. Franzosa E. A. Lloyd-Price J. McIver L. J. Schwager R. Poon T. W.. (2018). Dynamics of metatranscription in the inflammatory bowel disease gut microbiome. Nat. Microbiol. 3, 337–346. doi: 10.1038/s41564-017-0089-z 29311644 PMC6131705

[B48] Schirmer M. Stražar M. Avila-Pacheco J. Rojas-Tapias D. F. Brown E. M. Temple E.. (2024). Linking microbial genes to plasma and stool metabolites uncovers host-microbial interactions underlying ulcerative colitis disease course. Cell Host Microbe 32, 209–226. doi: 10.1016/j.chom.2023.12.013 38215740 PMC10923022

[B49] Segal J. P. Leblanc J.-F. Hart A. L. (2021). Ulcerative colitis: an update. Clin. Med. 21, 135–144. doi: 10.7861/clinmed.2021-0080 PMC8002778 33762374

[B50] Shen Z.-H. Zhu C.-X. Quan Y.-S. Yang Z.-Y. Wu S. Luo W.-W.. (2018). Relationship between intestinal microbiota and ulcerative colitis: Mechanisms and clinical application of probiotics and fecal microbiota transplantation. World J. Gastroenterol. 24, 5–14. doi: 10.3748/wjg.v24.i1.5 29358877 PMC5757125

[B51] Tong J. Liu C. Summanen P. Xu H. Finegold S. M. (2011). Application of quantitative real-time PCR for rapid identification of Bacteroides fragilis group and related organisms in human wound samples. Anaerobe 17, 64–68. doi: 10.1016/j.anaerobe.2011.03.004 21439390

[B52] Travis S. P. L. Higgins P. D. R. Orchard T. van der Woude C. J. Panaccione R. Bitton A.. (2011). Review article: defining remission in ulcerative colitis. Aliment Pharmacol. Ther. 34, 113–124. doi: 10.1111/apt.2011.34.issue-2 21615435

[B53] Wang R. Li Z. Liu S. Zhang D. (2023). Global, regional and national burden of inflammatory bowel disease in 204 countries and territories from 1990 to 2019: A systematic analysis based on the Global Burden of Disease Study 2019. BMJ Open 13, 1–15. doi: 10.1136/bmjopen-2022-065186 PMC10069527 36977543

[B54] Wang K. Liao M. Zhou N. Bao L. Ma K. Zheng Z.. (2019). Parabacteroides distasonis Alleviates Obesity and Metabolic Dysfunctions *via* Production of Succinate and Secondary Bile Acids. Cell Rep. 26, 222–235.e5. doi: 10.1016/j.celrep.2018.12.028 30605678

[B55] Wu X. Zhang T. Zhang T. Park S. (2024). The impact of gut microbiome enterotypes on ulcerative colitis: identifying key bacterial species and revealing species co-occurrence networks using machine learning. Gut Microbes 16, 1–20. doi: 10.1080/19490976.2023.2292254 PMC10761161 38117560

[B56] Zheng M. Han R. Yuan Y. Xing Y. Zhang W. Sun Z.. (2023). The role of Akkermansia muciniphila in inflammatory bowel disease: Current knowledge and perspectives. Front. Immunol. 13. doi: 10.3389/fimmu.2022.1089600 PMC9853388 36685588

